# The mode of subunit addition regulates the processive elongation of actin filaments by formin

**DOI:** 10.1016/j.jbc.2024.108071

**Published:** 2024-12-10

**Authors:** Biswaprakash Mahanta, Naomi Courtemanche

**Affiliations:** Department of Genetics, Cell Biology and Development, University of Minnesota, Minneapolis, Minnesota, USA

**Keywords:** formin, actin, profilin, processivity, fluorescence, microscopy

## Abstract

Formins play crucial roles in actin polymerization by nucleating filaments and regulating their elongation. Formins bind the barbed ends of filaments *via* their dimeric FH2 domains, which step processively onto incoming actin subunits during elongation. Actin monomers can bind formin-bound barbed ends directly or undergo diffusion-mediated delivery through interactions with formin FH1 domains and profilin. Despite its fundamental importance, a clear mechanism governing processive FH2 stepping has remained elusive. In this study, we systematically characterized the polymerization behavior of the *Saccharomyces cerevisiae* formin Bni1p using *in vitro* reconstitution assays and stochastic simulations. We found that Bni1p assembles populations of filaments with lengths that depend nonlinearly on the rate of elongation. This processive behavior is dictated by a variable probability of dissociation that depends on the reaction conditions. Bni1p dissociates from barbed ends with a basal off-rate, which enables prolonged filament assembly over the course of a long lifetime at the barbed end. A bias toward FH1-mediated delivery as the dominant mechanism for polymerization curtails elongation by shortening the lifetime of the formin at the filament end. This facilitates the assembly of populations of filaments with similar average lengths, even when polymerization proceeds at different rates. Our results suggest a central role for formin FH1 domains in regulating processivity. The specific effects of FH1 domains on processivity are variable and likely tailored to the physiological function of each formin.

The actin cytoskeleton is a dynamic network that drives cellular growth, motility, and division. In support of these functions, the formin family of proteins directs the polymerization of actin filaments that are incorporated into complex structures including filopodia, stress fibers, actin cables, and cytokinetic rings (1, 2). Formins bind actin filaments *via* their formin homology (FH) 2 domains, which associate into donut-shaped homodimers that encircle the barbed end (3, 4). During filament elongation, each FH2 domain within the dimer takes alternating steps onto incoming monomers to incorporate them into the growing filament (5, 6).

In cells, a substantial pool of actin monomers is bound by the abundant cytoplasmic protein profilin (7). Actin monomers and profilin–actin complexes can both bind formin-bound barbed ends directly (8, 9) (Fig. 1*A*). Profilin can also interact with polyproline tracts encoded in formin FH1 domains (10). The flexible nature of the FH1 domain facilitates diffusion-limited delivery of profilin–actin complexes to the barbed end (Fig. 1*A*) (11). Rapid dissociation of profilin from newly incorporated subunits regenerates the barbed end’s actin-binding site (12, 13), enabling polymerization to occur at rates exceeding those observed for filaments in the absence of formin (8).

**Figure 1:**
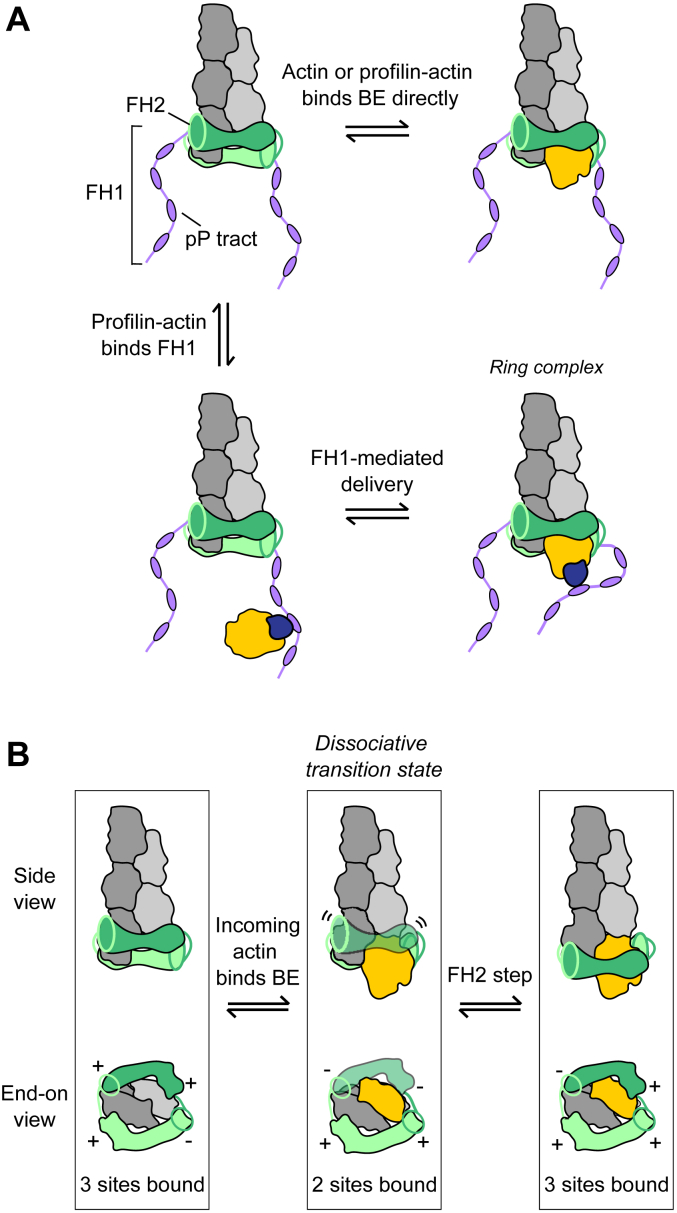
**FH2 stepping enables processive filament elongation.***A*, schematic representation of the two routes for actin subunits to bind a formin-bound barbed end. The actin filament is shown in *gray* and the FH2 dimer is shown in *green*. *Top*, an incoming actin monomer (*yellow*) or profilin–actin complex (not shown) can bind the barbed end directly. *Bottom*, a profilin–actin complex (*dark blue and yellow*) can bind a polyproline tract (pP tract; *purple ovals*) encoded in the FH1 domain for delivery to the barbed end. *B*, schematic representation of FH2 stepping. For clarity, the FH1 domains and profilin are not depicted. *Left*, the FH2 dimer binds the terminal subunits at the barbed end through engagement of three of its four actin-binding sites (3). Engagement of an actin-binding site is represented by “+”, and the free binding site is represented by “−”. *Center*, following binding of an incoming actin monomer, the FH2 dimer populates a dissociative transition state in which only two of its actin-binding sites engage the filament (3, 10). *Right*, the trailing FH2 domain (*dark green*) undergoes a translocation (*i.e.*, FH2 step) in the direction of the barbed end to bind the newly added actin subunit. FH2 stepping restores the number of engaged actin-binding sites to 3. FH, formin homology.

The process of FH2 stepping allows formins to persist at the barbed end through repeated cycles of subunit addition. This property is called processivity, and it enables formins to polymerize filaments that attain specific lengths while protecting barbed ends from capping and depolymerization (14, 15). The processive properties of formins vary widely. Some formins dissociate shortly following filament nucleation, whereas others facilitate the polymerization of thousands of actin subunits prior to dissociation (16, 17, 18, 19). The intrinsic processivity of each formin can also be modulated by physiological factors, including ionic strength, force, and interactions with formin-binding partners (17, 20, 21).

The process of stepping requires detachment of the trailing FH2 domain from its initial binding site to facilitate its translocation and the formation of stable contacts with a newly added actin subunit (Fig. 1*B*) (3). Consistent with the transient decrease in the number of contacts with the barbed end, FH2 dimers are thought to populate a transition state while stepping that promotes their dissociation from the barbed end (10).

Despite its fundamental importance to the polymerization activities of formins, a clear understanding of the mechanism governing processive FH2 stepping has remained elusive. The *Saccharomyces cerevisiae* formin Bni1p has been observed to dissociate from barbed ends at faster rates as the filament elongation rate increases (10). Based on these results, an initial model for formin processivity postulated that a formin’s association with the barbed end is regulated by the rate at which its FH2 domain steps onto incoming subunits. A subsequent study of the mammalian formins mDia1 and mDia2 revealed that dissociation of these formins from filament ends slows in the presence of profilin despite an increased rate of filament elongation (17). Thus, engagement of the FH1 domain to deliver profilin–actin complexes stabilizes these formins at barbed ends, independent of the rate of FH2 stepping. In addition to the central roles of the FH1 and FH2 domains, interactions between the C-terminal “tail” regions of formins with the sides of actin filaments further refine the processive behavior of formins (18, 19).

To generate a unified model for processivity that elucidates the roles of the FH1 and FH2 domains, we systematically characterized the polymerization behavior of Bni1p using *in vitro* reconstitution assays and stochastic simulations. This formin is highly processive and elongates filaments at rates that are readily modulated by changes in reaction conditions, making it an ideal candidate for this analysis. We found that Bni1p assembles populations of filaments with lengths that depend nonlinearly on the rate of elongation. This complex processive behavior is dictated by a variable probability of dissociation that depends on the reaction conditions. Bni1p dissociates from barbed ends with a basal off-rate, which enables prolonged filament assembly over the course of its long lifetime at the barbed end. A bias toward FH1-mediated delivery as the dominant mechanism for polymerization increases Bni1p′s dissociation rate, thereby curtailing elongation by shortening the lifetime of the formin at the filament end. This mechanism ultimately facilitates the assembly of populations of filaments with similar average lengths in conditions that favor FH1-mediated delivery, even when polymerization proceeds at different rates. Our results suggest a central role for formin FH1 domains in regulating processivity. The specific effects of the FH1 domain on processivity are variable and likely tailored to the physiological function of each formin.

## Results

To characterize the mechanism of formin processivity, we used total internal reflection fluorescence (TIRF) microscopy to visualize actin filaments as they are assembled by the *S. cerevisiae* formin Bni1p in the presence of profilin. To distinguish filament segments polymerized by Bni1p from those assembled spontaneously, we used actin monomers that were fluorescently labeled at cysteine 374 with Oregon Green 488 (22). This residue is located on the barbed end surface of the actin monomer, which provides the site for profilin binding (23). As a result, conjugation of a label to this residue reduces the binding affinity of actin for profilin 10-fold (24).

**Figure 2:**
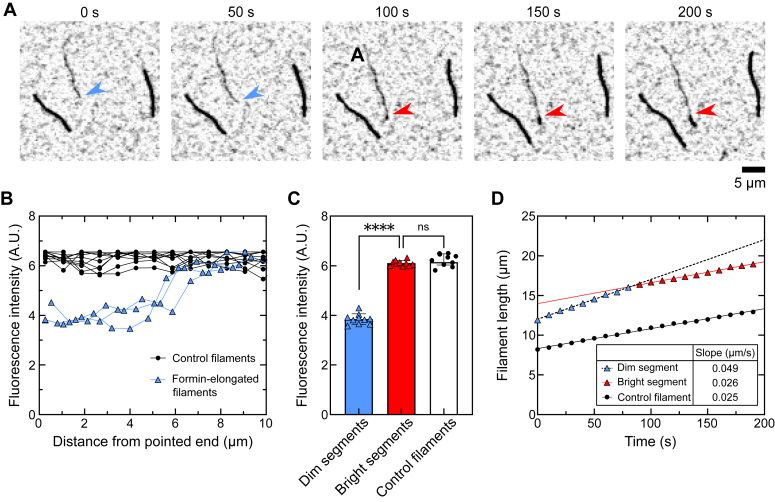
**Bni1p mediates processive filament assembly.***A*, time series of inverted fluorescence micrographs depicting the polymerization of one Bni1p-bound filament (*dim filament*) and two filaments with free barbed ends (*bright filaments*) in the presence of 0.75 μM actin monomers (33% Oregon Green-labeled), 15 nM Bni1p FH1FH2, and 5 μM *Saccharomyces cerevisiae* (*Sc*) profilin. The *blue arrowheads* point to the barbed end of the filament as it is elongated by Bni1p, and the *red arrowheads* point to the location at which the fluorescence intensity of the filament increases abruptly. The scale bar represents 5 μm. *B*, fluorescence intensity along the lengths of three representative control (*black*) and formin-assembled filaments (*blue*) as a function of the distance from their pointed ends. *C*, average fluorescence intensity along the lengths of control filaments (*white bar*) and dim and bright segments of formin-assembled filaments (*blue and red bars*). Error bars are standard deviations of the average fluorescence intensity measured for at least 10 filaments or filament segments. ∗∗∗∗ corresponds to *p* < 0.0001 and “n.s.” indicates *p* > 0.05 as determined using a one-way ANOVA test (F(2,27) = 480, *p* < 0.0001), followed by Tukey’s test (*p* < 0.0001 and *p* = 0.8539). *D*, change in filament length over time for a representative formin-bound filament and a filament with a free barbed end. *Blue and red triangles* correspond to length measurements preceding and following a change in the fluorescence intensity of the formin-assembled filament. Separate linear fits were applied to the change in length for each filament segment. The slopes of these linear fits are summarized in the inset. FH, formin homology.

We visualized filament elongation in reactions that included a 1:2 mixture of labeled and unlabeled actin monomers, a construct encoding the FH1 and FH2 domains of Bni1p, and *S. cerevisiae* profilin. In these conditions, labeled and unlabeled actin monomers bind free (*i.e.*, not formin-bound) filament barbed ends at equivalent rates, giving rise to filaments that are brightly and uniformly fluorescent along their lengths (22). Owing to the tighter affinity of profilin for unlabeled actin, a relatively small fraction of the profilin-bound actin is fluorescent. As a result, FH1-mediated delivery of profilin–actin results in a biased incorporation of unlabeled actin subunits into filaments assembled by formins. As previously reported, these filaments are more dimly fluorescent than filaments that are not polymerized by formin (Fig. 2*A*) (8, 9, 10).

We identified filaments assembled by Bni1p by comparing their fluorescence intensities to those of internal “control” filaments that polymerized spontaneously (Fig. 2*B*). We used time-lapse imaging to measure filament growth. Consistent with published studies, Bni1p-bound filaments elongated approximately twice as fast as control filaments in the presence of 5 μM profilin (8, 9, 10, 25). Over the course of our reactions, we observed an abrupt increase in fluorescence at the barbed ends of many formin-bound filaments (Fig. 2, *A* and *B*). The signal intensity measured along the lengths of the bright filament segments differed significantly from the fluorescence of the dim stretches and matched that of control filaments (Fig. 2, *B* and *C*). These brighter filament segments also elongated at a rate matching that of control filaments (Fig. 2*D*). Together, these observations indicate that transitions from dim to bright fluorescence intensities correspond to formin dissociation events at the barbed end. Quantification of the lengths of dim filament segments therefore provides a measure of the number of steps taken by Bni1p prior to its dissociation.

### Bni1p′s run length depends nonlinearly on the rate of elongation

FH2 dimers have been shown to adopt asymmetric conformations that encircle and bind the terminal subunits at filament barbed ends through engagement of 3 of their 4 actin-binding sites (Fig. 1*B*) (3). Binding of an incoming actin monomer to the unoccupied actin-binding site of an FH2 dimer has been proposed to promote detachment of the trailing FH2 domain, which then undergoes a translocation step to engage the new subunit. Consistent with a transient reduction in the number of occupied actin-binding sites from 3 to 2, FH2 stepping is thought to proceed through a dissociative transition state that promotes the release of the formin from the filament end (Fig. 1*B*) (10). The frequency with which the formin populates the dissociative state increases with the frequency of subunit addition, suggesting that the filament elongation rate may regulate the lengths of filaments assembled by formins prior to their dissociation.

We assessed the relationship between processivity and the rate of FH2 stepping by measuring the lengths of filaments polymerized by Bni1p over the course of our reactions. To minimize bias in our analysis, we only included filaments that contained two visible ends and a transition from dim to bright fluorescence. To confirm that fluorescent transitions correspond to formin dissociation events, we verified filament polarity by observing elongation for a minimum of 100 s. Although dim filaments lacking a transition to bright fluorescence were not included in our analysis, the lengths of these filaments measured at the end of our polymerization reactions fell within the range of lengths of dim segments of filaments from which a formin was observed to dissociate. Therefore, omission of filaments that are continually polymerized by formin does not bias our measurements to shorter lengths.

**Figure 3:**
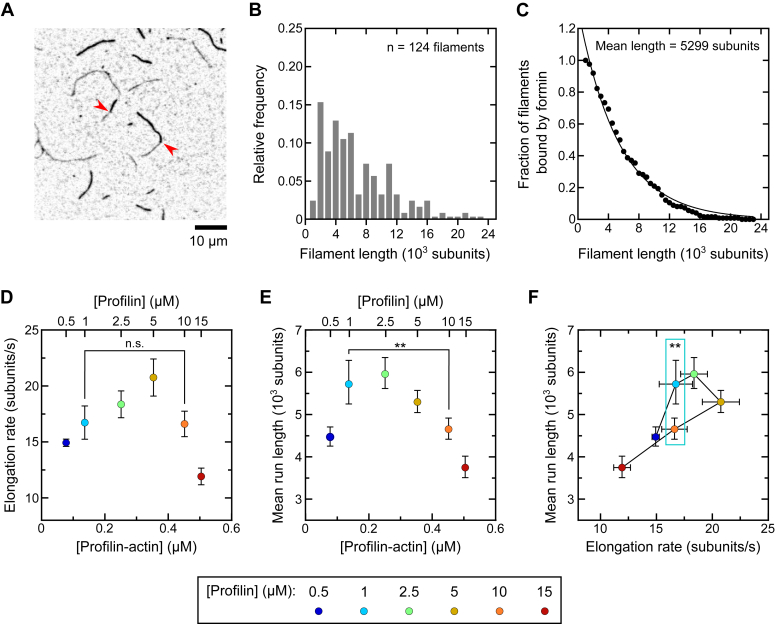
**Bni1p′s processivity depends nonlinearly on the rate of subunit addition.** The experimental conditions were as follows: 0.75 μM actin monomers (33% Oregon Green-labeled) in microscopy buffer with 4 to 35 nM Bni1p FH1FH2 and a range of concentrations of *Sc* profilin. The data were collected by TIRF microscopy. *A*, representative inverted fluorescence micrograph collected in the presence of 15 nM Bni1p and 5 μM profilin. The *red arrowheads* indicate positions at which formins have dissociated from filaments. The scale bar represents 10 μm. *B*, histogram of formin run lengths measured in the presence of 5 μM profilin. *C*, fraction of filaments bound by Bni1p as a function of length, obtained from four independent experiments performed in the presence of 5 μM profilin. A single-exponential fit was applied to the data to obtain the mean run length. *D*, dependence of the rate of filament elongation mediated by Bni1p on the concentrations of profilin-actin and profilin. Error bars are SDs of the mean elongation rate measured for at least 10 filaments. “n.s.” indicates *p* > 0.05 as determined using a one-way ANOVA test (F(5,54) = 62.7, *p* < 0.0001), followed by Tukey’s test (*p* = 0.9999). *E*, dependence of Bni1p′s run length on the concentration of profilin-actin and profilin. Error bars are the 95% confidence intervals of the mean formin run length measured from at least 70 filaments visualized in at least three independent experiments. ∗∗ indicates *p* < 0.01 as determined using a Kruskal–Wallis test (H (5) = 29.34, *p* < 0.0001), followed by Dunn’s test (*p* = 0.0063). *F*, dependence of Bni1p′s run length on the rate at which it elongates filaments. Data points are color-coded according to the concentration of profilin that was included in each reaction. Horizontal *error bars* are SDs of the mean elongation rates as measured in (*D*). Vertical *error bars* are the 95% confidence intervals of the mean formin run lengths as measured in (*E*). ∗∗ indicates *p* < 0.01 as determined using a Kruskal–Wallis test (H (5) = 29.34, *p* < 0.0001), followed by Dunn’s test (*p* = 0.0063). FH, formin homology; TIRF, total internal reflection fluorescence.

In the presence of 5 μM profilin, Bni1p assembled a population of filaments with differing lengths (Fig. 3*A*). The distribution of filament lengths—which correspond to the formin’s run lengths—is asymmetrical, and most filaments contain fewer than 6000 subunits (Fig. 3*B*). The distribution is well described by a single-exponential function (26, 27), with the exception of the first data point, which includes the filaments with the shortest lengths. Close inspection of our micrographs revealed that dim filament stretches containing 500 or fewer subunits are more difficult to quantify than longer stretches. This is consistent with a visible increase in the number of bright filaments assembled in reactions containing Bni1p compared to the number of filaments nucleated in control reactions lacking formin. Indeed, ∼6% of filaments initially identified as spontaneously-assembled in fact contain a dim stretch measuring 1 to 4 pixels in length at their pointed end (Fig. S1). As a result, the population of short run lengths is underrepresented in our histograms.

We plotted a survival curve depicting the fraction of filaments assembled by Bni1p that attain a specific length prior to dissociation of the formin (Fig. 3*C*). Application of a single-exponential function to the survival distribution yields the mean formin run length. To account for the underrepresentation of short run lengths in our experiments, we omitted the first data point when applying the fit. The y-intercept of the exponential fit is larger than 1, consistent with the underrepresentation of short formin run lengths in our experiments. In the presence of 5 μM profilin, this analysis produced a mean run length of ∼5300 subunits, consistent with previous studies that characterized Bni1p as a highly processive formin (10, 28).

To modulate the rate of FH2 stepping, we varied the concentration of profilin in our reactions. Profilin regulates formin-mediated elongation in a biphasic manner (Fig. 3*D*) (8). At subsaturating concentrations of profilin, the formation of profilin–actin complexes speeds elongation through engagement of the formin’s FH1 domain. When the concentration of profilin exceeds that of monomeric actin, competition for FH1 binding sites between profilin–actin complexes and free profilin slows formin-mediated elongation (5). As a result of this biphasic dependence on profilin, it is possible to generate similar elongation rates with different concentrations of profilin (Fig. 3*D*).

We observed a biphasic dependence of Bni1p′s run length on the concentration of profilin–actin (Fig. 3*E*). The maximal run length was generated at a lower concentration of profilin–actin than is required to produce the peak elongation rate. As a result, the run length does not depend linearly on the elongation rate (Fig. 3*F*). Notably, Bni1p assembles populations of filaments with significantly different average lengths (*p* < 0.01, as determined using a Kruskal–Wallis test) in the presence of 1 and 10 μM profilin, which promote similar elongation rates (Fig. 3, *D*–*F*). Therefore, the frequency of FH2 stepping does not directly dictate the formin’s run length.

### Profilin modulates Bni1p′s lifetime at the barbed end in a biphasic manner

**Figure 4:**
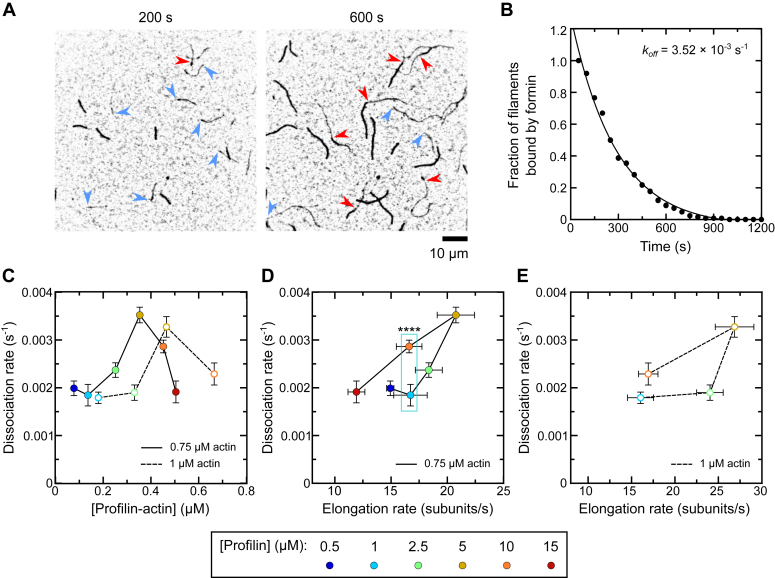
**Profilin modulates Bni1p′s lifetime at the barbed end in a biphasic manner.** The experimental conditions were as follows: 0.75 μM or 1 μM actin monomers (33% Oregon Green-labeled) in microscopy buffer with 2 to 35 nM Bni1p FH1FH2 and a range of concentrations of *Sc* profilin. The data were collected by TIRF microscopy. *A*, representative inverted micrographs collected 200 s and 600 s following the initiation of polymerization in the presence of 0.75 μM actin, 15 nM Bni1p, and 5 μM profilin. *Blue arrowheads* indicate the barbed ends of a representative set of formin-bound filaments. *Red arrowheads* indicate the positions at which formins have dissociated from the filaments. The scale bar represents 10 μm. *B*, fraction of filaments bound by Bni1p as a function of time, obtained from four independent experiments performed in the presence of 0.75 μM actin and 5 μM profilin. A single-exponential fit was applied to the data to obtain the dissociation rate (*k*_*off*_). *C*, dependence of the rate of Bni1p dissociation on the concentration of profilin-actin. *Filled and open circles* correspond to data collected in the presence of 0.75 and 1 μM actin. The error bars are the 95% confidence intervals obtained from exponential fits to survival plots as shown in (*B*). *D* and *E*, dependence of the rate of Bni1p dissociation on the rate of filament elongation in the presence of 0.75 μM (*D*) and 1 μM (*E*) actin. Data points are color-coded according to the concentration of profilin that was included in each reaction. Vertical error bars are the 95% confidence intervals for dissociation rates as calculated in (*C*). Horizontal error bars are SDs of the mean elongation rates measured for at least 10 filaments as calculated in Figure 3*D*. ∗∗∗∗ indicates *p* < 0.0001 as determined using an extra sum of squares F-test (F(1,21) = 73.56, *p* < 0.0001). FH, formin homology; TIRF, total internal reflection fluorescence.

A formin’s ability to incorporate actin subunits continuously into a growing filament depends on the lifetime of its association with the barbed end. This lifetime further dictates the cumulative amount of time the formin spends in the dissociative transition state that is populated upon FH2 stepping. To explore the temporal regulation of formin processivity, we calculated the time Bni1p spent at the barbed end of each filament prior to its dissociation by dividing the length of each dim filament stretch by the measured elongation rate (Fig. 4*A*). We plotted survival curves depicting the fraction of formin-assembled filaments that remain bound by Bni1p over time (Fig. 4*B*). Application of a single-exponential fit to each survival distribution generates a dissociation rate (*k*_*off*_) for the specific reaction condition (16, 17, 19). The dissociation rate measured in the presence of 5 μM profilin was ∼0.0035 s^−1^, consistent with previous measurements of Bni1p′s processivity (28).

As observed in our measurements of elongation rates and formin run lengths, varying the concentration of profilin modulates the dissociation rate in a biphasic manner (Fig. 4*C*). The concentration of profilin–actin required to produce the maximal dissociation rate depends on the actin concentration but coincides with the peak elongation rate (Fig. 4, *D* and *E*). At subsaturating concentrations of profilin, the dissociation rate is insensitive to changes in the reaction conditions. Therefore, although the fastest dissociation rates are observed in conditions producing the fastest elongation, the relationship between *k*_*off*_ and the rate of elongation is neither uniform nor linear (Fig. 4, *D* and *E*).

### A variable probability of dissociation dictates the processive behavior of Bni1p

**Figure 5:**
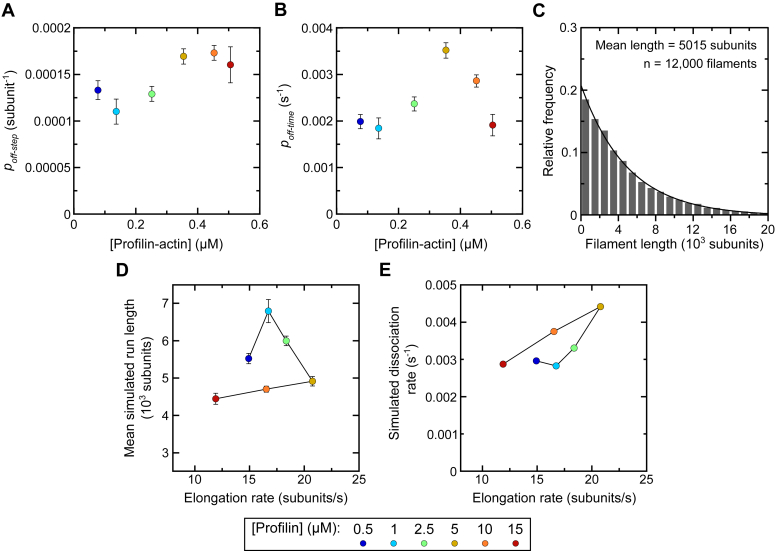
**A variable probability of dissociation dictates the processive behavior of Bni1p.***A*, dependence of the probability of Bni1p dissociation with each FH2 step (*p*_*off-step*_) on the concentration of profilin-actin. The probability values are calculated using survival curves depicting the fraction of filaments bound by Bni1p over time as depicted in Figure 4*B*, and the equation *p*_*off-step*_ = 1 – exp(-*k*_*off-step*_∗ Δ_step_), where *k*_*off-step*_ is the dissociation rate per FH2 step and Δ_step_ is an increment of 1 step. Single-exponential fits applied to the survival curves yield dissociation rates (*k*_*off-time*_), which were divided by the experimental elongation rate measured in each condition to obtain a dissociation rate per FH2 step (*k*_*off-step*_). Errors are calculated using the 95% confidence intervals obtained from the exponential fits used to determine the values of *k*_*off-time*_. Data points are color-coded according to the concentration of profilin that was included in each reaction. *B*, dependence of the probability of Bni1p dissociation per unit time (*p*_*off-time*_) on the concentration of profilin-actin. The probability values are calculated using survival plots depicting the fraction of filaments bound by Bni1p over time as depicted in Figure 4*B*, and the equation *p*_*off-time*_ = 1 – exp(-*k*_*off-time*_∗ Δ t), where *k*_*off-time*_ is the experimentally determined dissociation rate and Δ t is a time increment of 0.01 s. Errors are calculated using the 95% confidence intervals obtained from the exponential fits used to determine the values of *k*_*off-time*_. Data points are color-coded according to the concentration of profilin that was included in each reaction. *C*, histogram of formin run lengths generated in simulated polymerization reactions using the calculated value of *p*_*off-step*_ and the formin-mediated elongation rate derived from experimental reactions containing 0.75 μM actin and 5 μM profilin. A single-exponential fit was applied to the data to obtain the mean run length. *D*, dependence of the mean simulated formin run length obtained using calculated values of *p*_*off-step*_ on the rate of formin-mediated filament elongation. *Error bars* are SDs of the mean run lengths, obtained from 10 independent stochastic simulations. Data points are color-coded according to the concentration of profilin that was included in the experimental reactions used to measure the elongation rates. *E*, dependence of the simulated rate of formin dissociation on the rate of formin-mediated filament elongation. SDs of the dissociation rates were calculated from 10 independent stochastic simulations. The magnitude of each SD is smaller than the symbols used to represent the dissociation rates. Therefore, error bars are not visible in the graph. Data points are color-coded according to the concentration of profilin that was included in the experimental reactions used to measure the elongation rates. FH, formin homology.

The nonlinear dependence of Bni1p′s run length and dissociation rate on the filament elongation rate indicates that FH2 stepping does not uniformly regulate the association of Bni1p with barbed ends. This suggests that the probability that Bni1p will dissociate from the barbed end each time its FH2 domain steps onto an incoming actin monomer is variable and depends on the reaction conditions. To test this hypothesis, we determined the probability of Bni1p dissociation with each FH2 step (*p*_*off-step*_) using the survival distributions determined for each polymerization condition (Fig. 4*B*; see Experimental procedures). These probability values are indeed not constant and display a biphasic dependence on the concentration of profilin–actin (Fig. 5*A*).

We performed similar calculations to obtain the probability of formin dissociation per unit time (*p*_*off*-*time*_) (Fig. 4*B*). We found that the probability of dissociation of Bni1p from the barbed end over time also depends nonlinearly on the concentration of profilin–actin in each reaction (Fig. 5*B*).

To assess whether these variable probability measurements are sufficient to describe the processive behavior of Bni1p, we performed stochastic simulations of formin-mediated polymerization. Using our experimentally determined *p*_*off-step*_ values, we simulated the polymerization of 12,000 filaments over the course of a 20-min reaction by a formin whose processive behavior is dictated by a given probability of dissociation each time its FH2 domain steps onto an incoming actin subunit (see Experimental procedures). Consistent with our experimental observations, each simulated reaction produced a population of filaments with an exponential distribution of lengths (Fig. 5*C*). The average length of the filaments, obtained by fitting each distribution with a single-exponential function, depends nonlinearly on the filament elongation rate, as observed experimentally (Figs. 5*D* and 3*F*). We divided each simulated run length by the experimentally determined elongation rate to determine the lifetime of each formin–barbed end interaction. We used these lifetimes to generate survival plots from which we determined formin dissociation rates. These dissociation rates also display a nonlinear dependence on the elongation rate, matching the trend observed in our experiments (Figs. 5*E* and 4*D*). Similar simulations performed using our experimentally determined temporal probability parameters (*p*_*off-time*_) also produced mean run lengths and formin dissociation rates that depend nonlinearly on the filament elongation rate, closely replicating the trends observed in our experimental data (Fig. S2).

Taken together, our results reveal that Bni1p dissociates from filament ends with a variable probability that depends on the reaction conditions. The probability of dissociation with each FH2 step is sufficient to characterize the processive behavior of the formin. The lifetime of the formin’s association with the barbed end, which depends on the probability of dissociation over time, is also a key kinetic parameter that governs its processive behavior over a range of polymerization conditions.

### Bni1p′s lifetime at the barbed end is insensitive to the concentration of free profilin

To identify the origin of the variable probability with which Bni1p dissociates from barbed ends, we dissected our reaction conditions. Titration of profilin into reactions containing a constant concentration of actin monomers leads to the accumulation of both profilin–actin and free profilin. These molecular species compete to bind polyproline tracts encoded in formin FH1 domains, giving rise to the well-characterized biphasic dependence of the elongation rate on the concentration of profilin (Fig. 3*D*). It is possible that binding of free profilin to Bni1p′s FH1 domains might similarly influence Bni1p′s lifetime at the barbed end.

To test this possibility, we visualized Bni1p-mediated polymerization in the presence of human profilin-1 (*Hs* profilin). This profilin isoform binds actin with a nearly 30-fold tighter affinity than does *S. cerevisiae* (*Sc*) profilin (K_D_ = 0.1 and 2.9 μM, respectively) (29, 30). Therefore, similar levels of profilin–actin can be generated using lower concentrations of *Hs* profilin than *Sc* profilin, which also results in lower concentrations of free *Hs* profilin. We hypothesized that, if binding of free profilin to the FH1 domain regulates Bni1p′s lifetime at the barbed end, the use of lower concentrations of *Hs* profilin might produce a uniform, monophasic response in formin processivity to changes in the profilin concentration.

**Figure 6:**
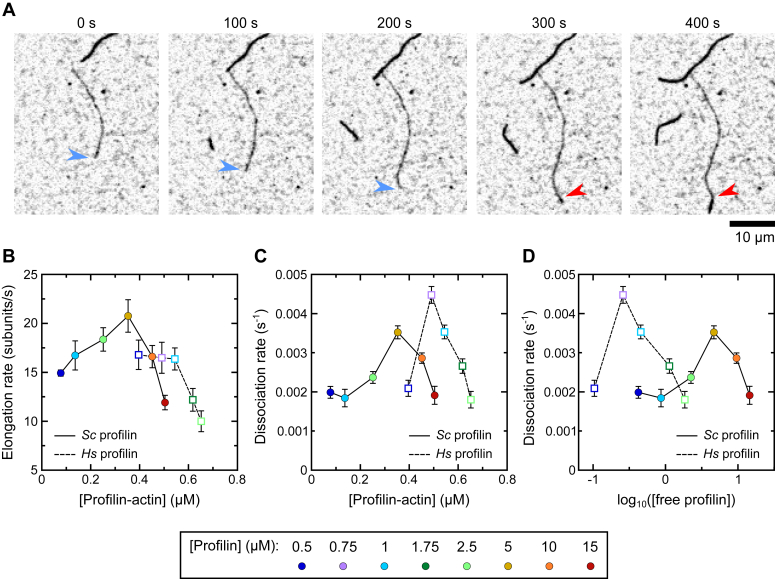
**Bni1p′s lifetime at the barbed end is insensitive to the concentration of free profilin.** The experimental conditions were as follows: 0.75 μM actin monomers (33% Oregon Green-labeled) in microscopy buffer with 4 to 60 nM Bni1p FH1FH2 and a range of concentrations of *Sc* or *Homo sapiens* (*Hs*) profilin. Data points are color-coded according to the concentration of profilin that was included in each reaction. *A*, time series of inverted fluorescence micrographs depicting the polymerization of one Bni1p-bound filament (*dim filament*) and two filaments with free barbed ends (*bright filaments*) in the presence of 40 nM Bni1p FH1FH2 and 1 μM *Hs* profilin. The *blue arrowheads* point to the barbed end of the filament as it is elongated by Bni1p, and the *red arrowheads* point to the location at which the fluorescence intensity of the filament increases abruptly. The scale bar represents 10 μm. *B*, dependence of the rate of filament elongation mediated by Bni1p on the concentrations of *Sc* (*circles*) and *Hs* (*squares*) profilin-bound actin. Rates obtained from reactions performed with *Sc* profilin-actin are identical to those depicted in Figure 3*D* and are included for comparison purposes. *Error bars* are SDs of the mean elongation rate measured for at least 10 filaments. *C*, dependence of the rate of Bni1p dissociation on the concentration of *Sc* (*circles*) and *Hs* (*squares*) profilin-bound actin. Rates obtained from reactions performed with *Sc* profilin-actin are identical to those depicted in Figure 4*C* and are included for comparison purposes. The error bars correspond to the 95% confidence intervals obtained from single-exponential fits of survival plots generated from measurements of at least 70 filaments visualized in at least three independent experiments. *D*, dependence of the rate of Bni1p dissociation on the concentration of free *Sc* (*circles*) and *Hs* (*squares*) profilin. Rates obtained from reactions performed using *Sc* profilin are identical to those depicted in Figure 4*C* and are included for comparison purposes. The error bars correspond to the 95% confidence intervals obtained from single-exponential fits of survival plots generated from measurements of at least 70 filaments visualized in at least 3 independent experiments. FH, formin homology.

We found that Bni1p mediates robust filament assembly in the presence of *Hs* profilin (Fig. 6*A*). In contrast to experiments performed with *Sc* profilin, the elongation rate is insensitive to changes in reaction conditions at concentrations of *Hs* profilin–actin below 0.6 μM (Fig. 6*B*). Elongation slows at higher concentrations of *Hs* profilin–actin. The fastest filament elongation rate we observed is 25% slower than the fastest rate measured in the presence of Bni1p′s cognate profilin, consistent with evolutionary co-optimization of the formin and profilin (31, 32).

Quantification of Bni1p dissociation rates revealed a biphasic dependence on the concentrations of both *Hs* profilin–actin and free *Hs* profilin (Fig. 6, *C* and *D*). We observed similar basal dissociation rates at concentrations of profilin below and above the peak in reactions containing *Hs* and *Sc* profilin. This suggests that the identity of the profilin isoform does not impact the intrinsic lifetime of the formin at the barbed end. The maximal dissociation rate is faster and occurs at higher concentrations of *Hs* profilin–actin and lower concentrations of free *Hs* profilin than in reactions containing *Sc* profilin. Thus, binding of free profilin to the FH1 domain does not give rise to the biphasic response in Bni1p′s processivity.

### FH1-mediated delivery regulates the dissociation of Bni1p from barbed ends

The lack of influence of free profilin on processivity suggests that the lifetime of Bni1p′s association with the barbed end might instead depend on interactions between profilin–actin and the formin’s FH1 domain. Consistent with this hypothesis, the mammalian formins mDia1 and mDia2 have been shown to dissociate more slowly from barbed ends in conditions that favor frequent FH1-mediated profilin–actin delivery (17). Adoption of the “ring complex” conformation, in which a profilin–actin complex is bound simultaneously to the barbed end and the FH1 domain (Fig. 1*A*), has therefore been proposed to stabilize formins at the ends of filaments.

**Figure 7:**
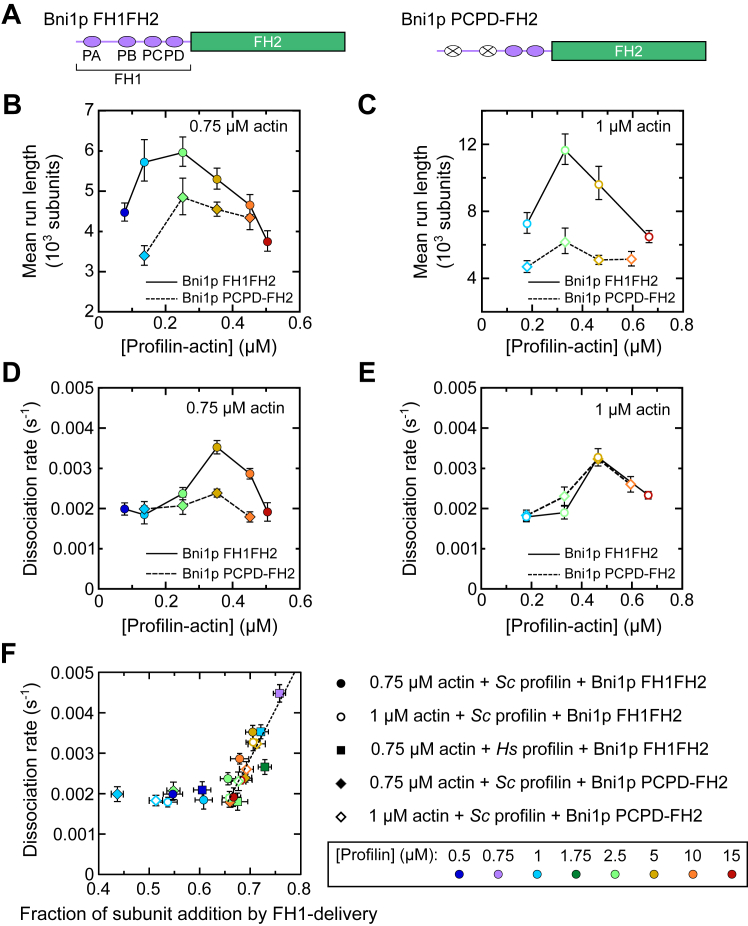
**FH1-mediated delivery regulates the dissociation of Bni1p from barbed ends.** The experimental conditions were as follows: 0.75 or 1 μM actin monomers (33% Oregon Green-labeled) in microscopy buffer with 4 to 35 nM Bni1p FH1FH2 or 7.5 to 35 nM Bni1p PCPD-FH2 and a range of concentrations of *Sc* profilin. Data points are color-coded according to the concentration of profilin that was included in each reaction. *A*, domain architecture of the Bni1p FH1FH2 and PCPD-FH2 constructs. Polyproline tracts are depicted as *purple ovals* and labeled profilin–actin, PB, *etc.* In the PCPD-FH2 construct, the profilin–actin and PB polyproline tracts are replaced with (Gly-Gly-Gly-Ser)_n_ sequences matching the length of each tract. *B* and *C*, dependence of the run lengths of Bni1p FH1FH2 (*circles*) and Bni1p PCPD-FH2 (*diamonds*) on the concentration of profilin-actin measured in the presence of (*B*) 0.75 μM and (*C*) 1 μM actin. Run lengths obtained from reactions performed with Bni1p FH1FH2 and 0.75 μM actin are identical to those depicted in 3E and are included for comparison purposes. Error bars are 95% confidence intervals obtained from single-exponential fits of survival plots generated from measurements of at least 70 filaments visualized in at least three independent experiments, as shown in Figure 3*B*. *D* and *E*, dependence of the rate of dissociation of Bni1p FH1FH2 (*circles*) and Bni1p PCPD-FH2 (*diamonds*) on the concentration of profilin-actin measured in the presence of (*B*) 0.75 μM and (*C*) 1 μM actin. Rates obtained from reactions performed with Bni1p FH1FH2 are identical to those depicted in Figure 4*C* and are included for comparison purposes. The error bars are the 95% confidence intervals obtained from single-exponential fits to survival plots generated from measurements of at least 70 filaments visualized in at least three independent experiments, as shown in Figure 4*B*. *F*, dependence of the rate of dissociation of Bni1p on the fraction of actin subunits that are polymerized *via* FH1-mediated delivery. The plot summarizes measurements performed in a range of experimental conditions, including 0.75 μM and 1 μM actin, *Sc* and *Hs* profilin, and using Bni1p FH1FH2 and Bni1p PCPD-FH2. A linear fit was applied to the data for which the fraction of actin polymerized by the FH1 domain exceeds two-thirds. The slope of the fit is 0.02 s^−1^. Horizontal *error bars* are SDs of the mean fraction of actin subunits polymerized *via* FH1-mediated delivery measured for at least 40 filaments in at least three independent experiments. Vertical error bars are the 95% confidence intervals obtained from single-exponential fits to survival plots generated from measurements of at least 70 filaments visualized in at least three independent experiments, as shown in Figure 4*B*. FH, formin homology.

To assay for stabilization of Bni1p at barbed ends upon adoption of the ring complex conformation, we decreased the efficiency of FH1-mediated delivery. We visualized polymerization using a construct in which the two N-terminal polyproline tracts have been replaced with (Gly-Gly-Gly-Ser)_n_ sequences matching the lengths of each tract (Fig. 7*A*). This construct (called “Bni1p PCPD-FH2”) mediates slower filament elongation than the WT construct, consistent with less frequent profilin–actin delivery to the barbed end (9, 25). Bni1p PCPD-FH2 assembles populations of filaments with exponential length distributions. The average formin run lengths calculated from fits to survival distributions are shorter than those observed for WT Bni1p in most reactions and converge with the WT lengths at the highest concentrations of profilin–actin we tested (Fig. 7, *B* and *C*). Bni1p PCPD-FH2 also exhibits a biphasic dissociation rate. In the presence of 0.75 μM actin, the dissociation rate is less sensitive to the concentration of profilin–actin than is observed for WT Bni1p (Fig. 7*D*). The maximal dissociation rate is ∼50% slower than the WT peak and occurs at the same concentration of profilin–actin. In the presence of 1 μM actin, Bni1p PCPD-FH2 dissociates from filaments at similar rates to those observed for the WT protein (Fig. 7*E*). Thus, an increase in the actin concentration reduces the sensitivity of Bni1p′s lifetime at the barbed end to changes in its FH1 domain sequence.

The rates at which Bni1p PCPD-FH2 dissociates from barbed ends at subsaturating concentrations of profilin are insensitive to changes in profilin–actin and match the WT rates (Fig. 7, *D* and *E*). In these conditions, polymerization occurs *via* both FH1-mediated delivery and direct binding of actin to FH2-bound barbed ends (Fig. 1*A*) (9). This suggests that an increase in the relative engagement of the FH1 domain, which occurs at intermediate concentrations of profilin–actin, may speed dissociation of the formin from the barbed end. To test for this possibility, we quantified the fraction of actin subunits that are polymerized *via* FH1-mediated delivery in each reaction condition. We took advantage of the fact that the FH1 domain preferentially polymerizes unlabeled actin owing to profilin’s weakened affinity for actin monomers conjugated with a label at cysteine 374. In contrast, FH2 domains polymerize labeled and unlabeled monomers with equal probability. We quantified the fluorescence intensities of filaments assembled by Bni1p. We normalized these intensities to the fluorescence of spontaneously assembled filaments to determine the fraction of fluorescently labeled actin monomers polymerized by the formin. We then used these fluorescence measurements and the affinities of profilin for labeled and unlabeled actin monomers to calculate the fraction of subunits that is polymerized by the FH1 domain in each reaction condition (see Experimental procedures) (9, 24, 30).

Our analysis revealed a constant rate of Bni1p dissociation from barbed ends in conditions in which less than two-thirds of polymerized actin subunits are incorporated *via* FH1-mediated delivery (Fig. 7*F*). In these reactions, direct binding of actin to FH2-bound barbed ends contributes significantly to polymerization. In conditions in which more than two-thirds of polymerized subunits are delivered by the FH1 domain, the dissociation rate increases linearly as the relative efficiency of delivery increases. This trend is consistent for filaments assembled in a range of concentrations of actin, profilin, and profilin–actin. It is also unaffected by changes in the identity of the profilin isoform or the FH1 domain sequence. These results reveal that Bni1p dissociates from barbed ends with a basal dissociation rate, which ensures a long lifetime at the barbed end and enables prolonged filament assembly. Domination of the FH1-mediated mechanism for polymerization increases the probability with which Bni1p dissociates, thereby shortening its lifetime at the filament end and curtailing formin-mediated elongation.

### Bni1p′s tail domain speeds dissociation from barbed ends

**Figure 8:**
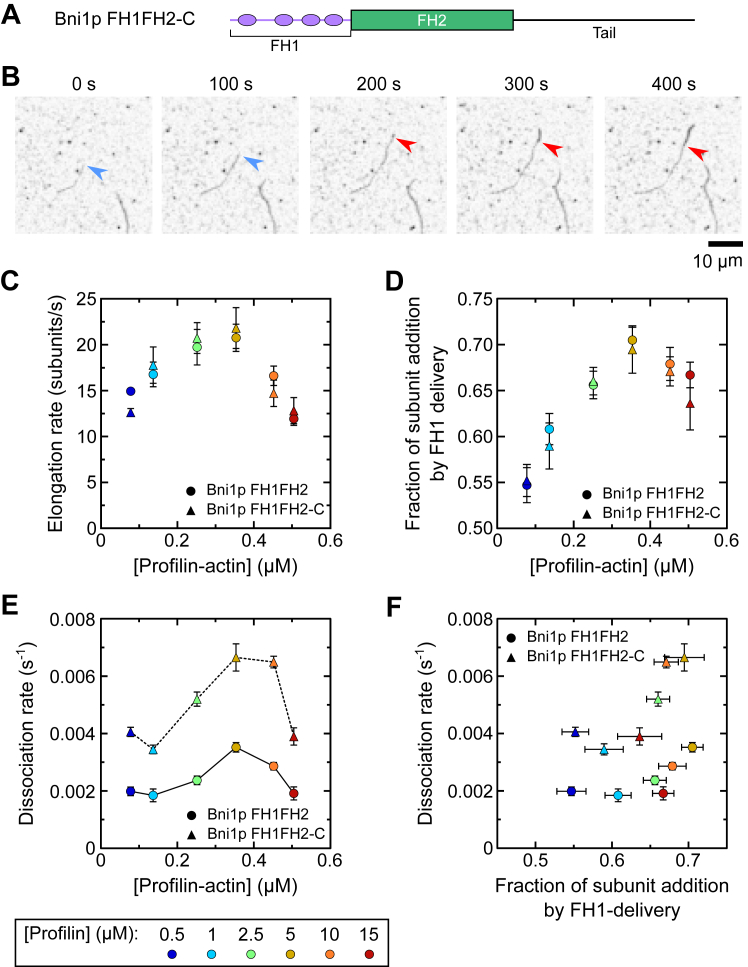
**Bni1p′s tail domain speeds dissociation from barbed ends.** The experimental conditions were as follows: 0.75 μM actin monomers (33% Oregon Green-labeled) in microscopy buffer with 4 to 35 nM Bni1p FH1FH2 or 5 to 85 nM Bni1p FH1FH2-C and a range of concentrations of *Sc* profilin. Data points are color-coded according to the concentration of profilin that was included in each reaction. *A*, domain architecture of the Bni1p FH1FH2-C construct. *B*, time series of inverted fluorescence micrographs depicting the polymerization of a Bni1p FH1FH2-C–bound filament (*dim filament*) in the presence of 5 μM *Sc* profilin. The *blue arrowheads* point to the barbed end of the filament as it is elongated by Bni1p, and the *red arrowheads* indicate the position at which the formin has dissociated from the filament. The scale bar is 10 μm. *C*, dependence of the rate of filament elongation mediated by Bni1p FH1FH2 (*circles*) and Bni1p FH1FH2-C (*triangles*) on the concentration of profilin-actin. Rates obtained from reactions performed with *Sc* profilin-actin are identical to those depicted in Figure 3*D* and are included for comparison purposes. The error bars are SDs of the mean elongation rate measured for at least 10 filaments. *D*, dependence of the fraction of actin subunits that are polymerized *via* FH1-mediated delivery by Bni1p FH1FH2 (*circles*) and Bni1p FH1FH2-C (*triangles*) on the concentration of profilin-actin. The error bars are SDs of the mean fraction of actin subunits polymerized *via* FH1-mediated delivery measured for at least 20 filaments in at least three independent experiments. *E*, dependence of the rate of dissociation of Bni1p FH1FH2 (*circles*) and Bni1p FH1FH2-C (*triangles*) on the concentration of profilin-actin. Dissociation rates obtained from reactions performed with Bni1p FH1FH2 are identical to those depicted in Figure 4*C* and are included for comparison purposes. The *error bars* are the 95% confidence intervals obtained from single-exponential fits to survival plots generated from measurements of at least 50 filaments visualized in at least three independent experiments, as shown in Figure 4*B*. *F*, dependence of the rate of dissociation of Bni1p FH1FH2 (*circles*) and Bni1p FH1FH2-C (*triangles*) on the fraction of actin subunits that are polymerized *via* FH1-mediated delivery. Dissociation rates obtained from reactions performed with Bni1p FH1FH2 are identical to those depicted in Figure 4*C* and are included for comparison purposes. Horizontal error bars are SDs of the mean fraction of actin subunits polymerized *via* FH1-mediated delivery as calculated in panel (*D*). Vertical error bars are the 95% confidence intervals obtained from single-exponential fits to survival plots generated from measurements of at least 50 filaments visualized in at least three independent experiments, as shown in Figure 4*B*. FH, formin homology.

Formin tail domains encompass the region between the FH2 domain and the C terminus of the protein. These regions vary widely in length and have been shown to enhance or inhibit processivity depending on their sequence (18). Some tail domains have been demonstrated to increase formin processivity up to 15-fold through electrostatic interactions with the sides of actin filaments (19). The magnitude of this effect suggests that tail domains could negate the role of FH1-mediated delivery in tuning formin processivity. To assess the physiological relevance of the impacts of the FH1 domain on processivity, we visualized filament elongation produced by a construct of Bni1p encoding its FH1, FH2, and C-terminal tail domains (called “Bni1p FH1FH2-C”) (Fig. 8*A*).

As reported previously, we found that Bni1p FH1FH2-C promotes efficient actin polymerization in the presence of a range of concentrations of profilin (Fig. 8*B*) (33). Bni1p FH1FH2 and Bni1p FH1FH2-C elongate filaments at the same rate in each reaction condition (Fig. 8*C*). The fraction of actin subunits that are polymerized *via* FH1-mediated delivery is also unaffected by the tail domain (Fig. 8*D*). Therefore, the tail domain does not influence the mechanism of filament elongation mediated by Bni1p.

The isoelectric point of Bni1p′s tail domain (pI = 5.21) nearly matches that of actin (pI = 5.29), suggesting that stabilizing electrostatic interactions between the tail and filament sides are unlikely. Consistent with this, we found that Bni1p FH1FH2-C dissociates from filament ends at a faster rate than does Bni1p FH1FH2 in each reaction condition (Fig. 8*E*). Despite this decrease in processivity, the dissociation rates observed for Bni1p FH1FH2-C retain a biphasic dependence on the concentration of profilin–actin. The net increase in the dissociation rate is approximately uniform across the range of concentrations of profilin–actin we sampled. Plotting the dissociation rate as a function of the fraction of subunits polymerized *via* FH1-mediated delivery reveals that Bni1p FH1FH2-C dissociates from filament ends at a constant rate when less than two-thirds of polymerized actin subunits are incorporated *via* FH1-mediated delivery (Fig. 8*F*). This rate is approximately two-fold faster than the basal dissociation rate measured for Bni1p FH1FH2. When more than two-thirds of polymerized subunits are delivered by the FH1 domain, the dissociation rate increases linearly as the efficiency of delivery increases. Thus, the FH1 domain plays a definitive role in modulating Bni1p′s processivity, both in the absence and the presence of its tail domain.

## Discussion

Formins regulate the elongation of actin filaments by processively stepping onto incoming subunits to incorporate them at the barbed end. To understand the mechanism underlying processivity, we observed actin polymerization mediated by the *S. cerevisiae* formin Bni1p. We found that Bni1p assembles populations of filaments with average lengths that depend nonlinearly on the rate of elongation. The rate at which Bni1p dissociates from barbed ends also depends nonlinearly on the rate of elongation. This processive behavior is dictated by a variable probability of dissociation that depends on the reaction conditions.

### Engagement of the FH1 domain regulates formin processivity in an isoform-dependent manner

To uncover the origin of the complex relationship between the rates of formin dissociation and filament elongation, we considered the contributions of each of the molecular species in our polymerization reactions. Changes in the concentrations of actin and free profilin do not eliminate the biphasic dependence of the dissociation rate on the concentration of profilin–actin complexes, ruling out binding of free profilin to the FH1 domain as the source for this behavior (Fig. 6*D*). The slowest dissociation rates observed in each set of reactions are similar, independent of the concentration of actin or profilin, or the identity of the profilin (Figs. 4*C*, and 6*C*). A variant of Bni1p in which the FH1 domain encodes only two polyproline tracts exhibits a similar minimum dissociation rate (Fig. 7*D*). This suggests that a basal rate of dissociation governs the interaction of Bni1p with filament ends, independent of reaction conditions.

The lifetime of the interaction of the Bni1p PCPD-FH2 variant with barbed ends is also less sensitive to changes in the concentration of profilin–actin than is observed for WT Bni1p (Fig. 7*D*). Thus, FH1-mediated elongation plays a mechanistic role in the dissociation of the formin from the barbed end. Consistent with this model, calculation of the fraction of actin subunits polymerized *via* FH1-mediated delivery in each reaction condition revealed a striking dependence of the dissociation rate on the mode of subunit addition employed by the formin (Fig. 7*E*).

**Figure 9:**
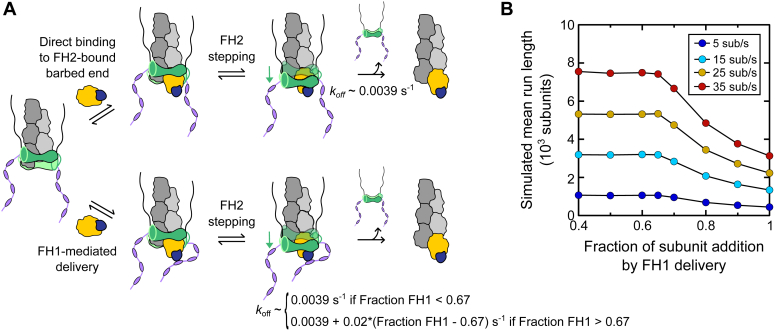
**Model for processive filament elongation by Bni1p.***A*, schematic representation of actin filament elongation mediated by the FH1 (*purple*), FH2 (*green*), and C-terminal tail (*black*) domains of Bni1p. *Top row*, when actin monomers (not shown) and profilin–actin complexes (*blue and yellow*) bind the FH2-bound barbed end directly, dissociation of the formin from the barbed end occurs at a basal rate of ∼0.0039 s^−1^. *Bottom row*, when the fraction of monomers polymerized *via* the FH1 domain is below two-thirds (*i.e.*, fraction FH1 < 0.67), the formin dissociates at the basal rate of 0.0039 s^−1^. When the fraction of monomers polymerized *via* the FH1 domain exceeds two-thirds (*i.e.*, fraction FH1 > 0.67), the dissociation rate increases by 0.02 multiplied by the fraction of monomers polymerized *via* the FH1 domain that exceeds two-thirds (*i.e.*, 0.0039 + 0.02∗(fraction FH1 – 0.67) s^−1^). *B*, dependence of mean simulated run lengths on the fraction of actin subunits that are polymerized *via* FH1-mediated delivery. Simulations were performed using filament elongation rates of 5 sub/s (*dark blue data*), 15 sub/s (*light blue data*), 25 sub/s (*yellow data*), and 35 sub/s (*red data*). SDs of the mean simulated run lengths were calculated from 10 independent simulations. The magnitude of each SD is smaller than the symbols used to represent the simulated run lengths. Therefore, *error bars* are not visible in the graph. FH, formin homology.

In conditions that promote polymerization through both FH1-mediated delivery and direct binding of actin to the FH2-bound barbed end, dissociation of Bni1p from the barbed end occurs at a constant, basal rate (Fig. 9*A*). Thus, Bni1p′s association at the barbed end is most stable when its FH2 domain steps frequently onto subunits that bind the barbed end directly. The lifetime of the formin–barbed end interaction shortens when FH1-mediated delivery becomes the dominant mode of subunit addition (Fig. 9*A*). In this regime, the rate of formin dissociation increases linearly rather than occurring at a higher, fixed value. This suggests that the probability that an incoming actin subunit is delivered by the FH1 domain is a key determinant of the rate of formin dissociation from filament ends.

Recent structures of formins bound to filament ends provide insights into a possible mechanism for promoting dissociation upon FH2 stepping. These structures revealed that profilin and the FH2 dimer share overlapping binding sites on the terminal actin subunit (3). Competition for occupancy of these binding sites has been proposed to promote dissociation of profilin from the formin-bound barbed end. However, a structure solved using a profilin variant that binds actin with unusually tight affinity revealed flexibility in the orientation of the FH2 dimer at the barbed end that might also promote dissociation of the formin (3). In this structure, the leading FH2 domain undergoes a rotation of ∼15° to accommodate profilin binding at the terminal subunit. This rotation displaces the FH2 domain away from its canonical binding site and might therefore weaken the formin’s association with the barbed end. Although the flexible FH1 domains are not resolved in this structure, it is conceivable that engagement of these molecular tethers, which physically bridge the FH2 domain and an incoming profilin–actin complex, may constrain the conformational dynamics of the leading FH2 domain during the delivery step. As a result, FH2 stepping onto FH1-delivered actin subunits may increase the probability of formin dissociation above the basal probability associated with stepping onto subunits that bind the barbed end directly (Fig. 9*A*).

What is the mechanistic significance of the two-thirds threshold value for FH1-mediated polymerization? At this value, the fraction of subunits delivered by the FH1 domain significantly exceeds the fraction of monomers that bind the barbed end directly, suggesting that the rate of formin dissociation does not depend simply on the likelihood of FH1-mediated delivery for each incoming subunit. We propose that destabilization of Bni1p at the barbed end occurs not when a single subunit is delivered by the FH1 domain, but rather when multiple delivery events occur in a succession. For example, when 67% of polymerized subunits are incorporated *via* the FH1 domain, the likelihood of two consecutive delivery events is nearly 50%. Beyond this threshold value, consecutive instances of FH1-mediated delivery become the dominant mode of subunit addition. Thus, whereas FH2 stepping may be insensitive to single instances of FH1-mediated delivery, stepping onto a second, FH1-delivered subunit may impose conformational constraints that promote dissociation of the formin from the barbed end.

The effects of FH1-mediated delivery on the processive behavior of Bni1p differ from the results of a study performed with the mammalian formins mDia1 and mDia2 (17). Although the FH1 domain regulates the processivity of all three formins, the lifetimes of mDia1 and mDia2 at the barbed end were shown to increase upon engagement of FH1-mediated delivery. Therefore, whereas modulation of processivity appears to be a unifying property of formin FH1 domains, the specific effect on the lifetime of the formin at the filament end is isoform-specific. Structural variations among formin isoforms provide a plausible explanation for this difference. For example, the FH2 dimer of mDia1 is ∼7% wider than those of the mammalian formin INF2 and the *S. pombe* formin Cdc12p (3). This additional width may confer conformational flexibility that increases the faithfulness of FH2 stepping onto profilin–actin subunits delivered by the FH1 domain.

C-terminal tail domains also influence the processive properties of formins in an isoform-specific manner. In contrast to the destabilizing effects of Bni1p′s tail domain (Fig. 8*E*), the tail regions of mDia1 and mDia2 have been shown to increase the lifetime of formin–barbed end interactions (19). The tails of mDia1 and mDia2 are both positively charged (pI = 9.59 and 9.86), so the enhancement in processivity likely arises from stabilizing electrostatic interactions with the sides of actin filaments (19). The magnitude of the increase in the dissociation rate produced by Bni1p′s tail is approximately uniform across a range of concentrations of profilin–actin (Fig. 8*E*). This is consistent with a model in which a formin’s tail influences the intrinsic processivity of its FH2 domain by either stabilizing or destabilizing the formin’s association with the barbed end. This basal processivity is then tuned by the frequency of FH1-mediated delivery during filament elongation.

The differences in the processive behaviors of Bni1p and mDia1 are consistent with the diverging responses of these formins to force. In the absence of profilin, tensile force speeds filament elongation mediated by mDia1 (17, 34, 35) but slows elongation by Bni1p (36). In contrast, elongation rates generated by both formins increase under force in the presence of profilin (34, 37, 38, 39). Thus, force modulates the interactions of formins with barbed ends in isoform-specific ways that either promote or inhibit the binding of actin monomers.

The specific effects of the FH1 domain on processivity are likely tailored to the physiological function of each formin. For example, mDia1 polymerizes filaments that are incorporated into stress fibers, which can span distances exceeding 15 μm in mouse embryonic fibroblasts (1). A long lifetime at the barbed end enables mDia1 to polymerize filaments that attain appropriate lengths while competing with capping and depolymerization factors (40, 41). Speckles of fluorescently labeled mDia1 molecules have been observed to move directionally for at least 5 μm in live cells (42, 43), consistent with processive polymerization. In contrast, Bni1p localizes transiently to the *S. cerevisiae* bud tip, where it polymerizes filaments that are incorporated into actin cables measuring up to 6 μm (44, 45). Visualization of actin cables in fission yeast revealed that these structures are composed of individual actin filaments measuring 0.4 to 0.5 μm in length (46). A computational model assuming individual filament lengths of 0.5 μm successfully recapitulates budding yeast actin cable assembly, thus supporting a requirement for shorter Bni1p run lengths than mDia1 in cells (47).

### Filament lengths converge when FH1-mediated delivery is maximally efficient

A substantial pool of actin monomers is bound by profilin in cells (7), making it likely that FH1-mediated delivery is the dominant mechanism for formin-mediated elongation (9). To investigate how this mode of subunit addition would impact Bni1p′s processivity, we simulated polymerization generated by a formin employing combinations of FH1-mediated delivery and direct subunit binding that extend beyond the experimentally accessible limits of our reactions. In simulations sampling polymerization below the two-thirds threshold in FH1 efficiency, we employed the average basal dissociation rate displayed by Bni1p FH1FH2-C (*i.e.*, 0.0039 s^−1^) to calculate the probability of formin dissociation (Fig. 8*E*). We applied a linear fit to our experimental data to extrapolate dissociation rates in conditions in which more than two-thirds of the polymerized actin subunits are incorporated *via* FH1-mediated delivery (Fig. 7*E*). We used the dissociation rates measured in reactions containing Bni1p FH1FH2 for this fit because the large number of data points facilitates an accurate fit that is also consistent with the behavior of Bni1p FH1FH2-C. The slope of this fit is 0.02 s^−1^, indicating that an increase of 10% in the relative contribution of FH1-mediated delivery increases the dissociation rate by ∼50%. We used these extrapolated dissociation rates to calculate probabilities of formin dissociation per unit time.

At each elongation rate we sampled, our simulations reveal a constant formin run length when fewer than two-thirds of the polymerized subunits are incorporated *via* the FH1 domain (Fig. 9*B*). In these conditions, the average length of the assembled filaments is therefore insensitive to variations in the efficiency of FH1-mediated delivery. Above this threshold efficiency, the average run length of the formin decreases and attains a minimum value when FH1-mediated delivery is the only polymerization mechanism employed. Increasing the formin’s elongation rate produces longer run lengths. However, the differences in the average run lengths generated across the range of elongation rates decrease when FH1-mediated delivery becomes the dominant mode of polymerization (Fig. 9*B*).

Our simulations show that when at least one-third of polymerized subunits are incorporated into filaments *via* direct binding to the barbed end, the average length of the assembled filaments depends directly on the elongation rate. On the other hand, a bias toward FH1-mediated delivery facilitates the assembly of populations of filaments with similar lengths, even when polymerization proceeds at different rates. This mechanism ultimately minimizes variations in formin elongation activity in favor of filament network uniformity.

### Complex mechanisms tune formin processivity in cells

Beyond the crucial role of the FH1 domain, other physiological factors further modulate the processive behaviors of formins. Electrostatic interactions play important regulatory roles in stabilizing formin FH2 dimers at barbed ends (17, 48). Processivity is differentially influenced by electrostatic interactions between the C-terminal tail domains of formins and the sides of elongating filaments (19). Depending on the formin, the contributions of the tail domains to processivity can be sequence-specific (18). Formins and capping protein have also been observed to colocalize at barbed ends, where they form short-lived “decision complexes” that increase the rate of dissociation of both the formin and capping protein from the barbed end (40, 41). In *S. cerevisiae*, the cell polarity factor Bud14p binds the formin Bnr1p and promotes its displacement from barbed ends (20). Piconewton forces also dramatically attenuate the processivity of mDia1 and mDia2 (17). Formin-mediated polymerization is further regulated through synergistic interactions with other cytoskeletal proteins, including Spire (49), Ena/VASP (50), adenomatous polyposis coli (51, 52), and CLIP170 (53). A complete understanding of the mechanisms governing processive polymerization by formins will require the integration of all of these factors.

## Experimental procedures

### Protein purification

A construct encoding the FH1 and FH2 domains of the *S. cerevisiae* formin Bni1p (“Bni1p FH1FH2”; residues 1227–1776) was cloned into a pGEX-4T-3 plasmid (GE Healthcare Life Sciences). A second construct included the formin’s FH1, FH2, and C-terminal tail domains (“Bni1p FH1FH2-C”; residues 1227–1953). In addition to an N-terminal glutathione S-transferase (GST) tag, both constructs encoded an N-terminal tobacco etch virus (TEV) protease recognition sequence and a C-terminal 6 × His tag. To generate the Bni1p PCPD-FH2 variant, the two N-terminal polyproline tract sequences located in the FH1 domain of the Bni1p FH1FH2 construct were replaced with a repetitive (Gly-Gly-Gly-Ser)_n_ sequence of the same length. All three constructs were expressed at 16 °C overnight in 1-l cultures of BL21(DE3) RP Codon Plus cells (Agilent Technologies). Unless otherwise stated, all chemicals were purchased from Sigma-Aldrich.

Resuspended cell pellets were sonicated in lysis buffer (50 mM Tris (pH 8.0), 500 mM NaCl, and 1 mM DTT), and centrifuged at 20,100*g* for 40 min. The supernatant was incubated with glutathione-Sepharose resin (Gold Biotechnology) with rotation for 1 h at 4 °C and transferred into an empty glass column. The resin was washed with lysis buffer, low-salt wash buffer (50 mM Tris (pH 8.0), 100 mM NaCl, and 1 mM DTT), and eluted with 100 mM GSH (pH 8.0) in low-salt wash buffer. The eluted protein was incubated with ∼2 to 5 μM MBP-tagged TEV protease overnight at 4 °C to remove the GST tag. Purified proteins were separated from the TEV protease and cleaved GST by nickel affinity chromatography, concentrated using 30,000 molecular-weight cut-off spin columns (EMD Milipore), and dialyzed into KMEI buffer (50 mM KCl, 1 mM MgCl_2_, 1 mM EGTA, and 10 mM imidazole (pH 7.0)) with 1 mM DTT. The dialysis buffer for Bni1p FH1FH2-C also contained 5% glycerol. Purified formins were flash-frozen and stored at −80 °C. We used ProtParam (http://web.expasy.org/protparam) to calculate extinction coefficients (54).

*S. cerevisiae* and *Homo sapiens* profilin were expressed from pMW172 vectors in BL21(DE3)pLysS cells and purified as described previously (11, 39). We used extinction coefficients of 19,060 and 18,450 M^–1^ cm^–1^ at λ = 280 nm for *S. cerevisiae* and *H. sapiens* profilin, respectively. Skeletal muscle actin was purified from an acetone powder prepared from frozen chicken breasts (Trader Joe’s) by one cycle of polymerization and depolymerization (55). Actin was labeled on cysteine 374 with Oregon Green 488 maleimide (Thermo Fisher Scientific) (22). Monomers were gel-filtered on S-300 resin in G-buffer (2 mM Tris (pH 8.0), 0.2 mM ATP, 0.5 mM DTT, and 0.1 mM CaCl_2_) and stored at 4 °C. We used extinction coefficients of 26,000 M^–1^ cm^–1^ at λ = 290 nm for unlabeled actin and 78,000 M^–1^ cm^–1^ at λ = 491 nm for Oregon Green. We used the following equation to calculate the concentration of Oregon Green-labeled actin: [Total actin] (μM) = [*A*_290_ – (*A*_491_ × 0.171)]/26,000 M^–1^ cm^–1^.

### Microscopy and data analysis

Glass coverslips (22 mm × 50 mm) and slides were sonicated in 2% Hellmanex III solution, rinsed with water, and sonicated in water. Flow chambers were prepared as described previously (10). Assembled chambers were stored at room temperature and used within 1 week.

Chambers were prepared with sequential incubations with 0.5% Tween-20 in high-salt tris-buffered saline (TBS) (HS-TBS; 50 mM Tris (pH 7.5), 600 mM NaCl), ∼1 μM N-ethylmaleimide-inactivated chicken skeletal muscle myosin (NEM-myosin) in HS-TBS, and 100 mg/ml bovine serum albumin in HS-TBS (25). The chamber was washed with HS-TBS between each incubation step and with KMEI prior to the introduction of a polymerization reaction.

Mixtures of labeled and unlabeled Ca^2+^ ATP-actin were converted to Mg^2+^ ATP-actin by adding 50 μM MgCl_2_ and 0.2 mM EGTA. Polymerization was initiated by addition of 2x microscopy buffer (1x microscopy buffer: 10 mM imidazole, pH 7.0, 50 mM KCl, 1 mM MgCl_2_, 1 mM EGTA, 50 mM DTT, 0.2 mM ATP, 15 mM glucose, 20 μg/ml catalase, 100 μg/ml glucose oxidase, and 0.5% (w/v) methylcellulose (4000 cP at 2% (w/v)) with formin and profilin. Reactions were introduced into the chamber for imaging upon initiation of polymerization.

Time-lapse images of elongating actin filaments were collected by through-objective TIRF microscopy on an Olympus Ti83 motorized microscope equipped with a cellTIRF system using a 60x, 1.49 NA objective and a 488 nm laser. Images were acquired every 10 s using a Hamamatsu C9100-23B ImagEM X2 EMCCD camera and CellSens Dimension software (Olympus; https://www.olympus-lifescience.com/en/software/cellsens/). Each polymerization reaction was initially imaged over a period of 20 to 30 min (“long timelapse”). Shorter movies consisting of 10 to 20 frames (collected over 100–200 s) were also collected to increase the number of filaments for analysis, as described below.

Time-lapse movies were processed with ImageJ software (National Institutes of Health; https://imagej.net/software/fiji/) (56). For each reaction, changes in filament length over time were measured using at least 20 successive frames collected over a time span of 200 s. To determine elongation rates, linear fits were applied to plots of filament length over time using GraphPad Prism 8 software (Dotmetrics; https://www.graphpad.com/). We measured the fluorescence intensities of formin-bound and control filaments by performing line scans along the lengths of filaments and adjacent background regions using ImageJ. Each line scan was performed in triplicate and measurements were taken at multiple time points to avoid any influence of photobleaching.

### Calculation of profilin–actin concentrations

Concentrations of profilin–actin were calculated using published dissociation constants for *S. cerevisiae* profilin and human profilin-1 for unlabeled and labeled actin monomers (24, 29, 30). The concentration of *Sc* profilin-actin was calculated using the following equation (57):

(1) $$\left[PA\right]=\left[A\right]*\frac{\left[P_{free}\right]}{K_{d}*\left[P_{free}\right]}$$

where [*A*] is the concentration of actin monomers (0.5 μM unlabeled monomers and 0.25 μM labeled monomers for experiments containing 0.75 μM total actin; and 0.67 μM unlabeled monomers and 0.33 μM labeled monomers for experiments containing 1 μM total actin), [*P*_*free*_] is the free concentration of profilin in each reaction, and *K*_*d*_ is the affinity of profilin for actin monomers (2.9 μM and 29 μM, for unlabeled and labeled actin monomers, respectively) (24, 30). The total concentration of profilin–actin complexes was determined by calculating the sum of the concentrations of profilin-bound unlabeled actin and profilin-bound labeled actin.

The formation of *Hs* profilin–actin complexes is characterized by a dissociation constant that is tighter than the concentration of actin monomers used in our reactions. These concentrations were therefore calculated using the following equation (57):

(2) $$\left[PA\right]=\frac{\left(\left[P_{total}\right]+\left[A\right]+K_{d}\right)-\sqrt{{\left(\left[P_{total}\right]+\left[A\right]+K_{d}\right)}^{2}-4*\left[P_{total}\right]*\left[A\right]}}{2}$$

where [*A*] is the concentration of actin monomers (0.5 μM unlabeled monomers and 0.25 μM labeled monomers), [*P*_*total*_] is the total concentration of profilin included in each reaction, and *K*_*d*_ is the affinity of profilin for actin monomers (0.1 μM and 1.0 μM, for unlabeled and labeled actin monomers, respectively) (24, 29). The total concentration of profilin–actin complexes was determined by calculating the sum of the concentrations of profilin-bound unlabeled actin and profilin-bound labeled actin.

### Calculations of formin run lengths and off-rates

To generate a large filament sample size for calculations of formin run lengths and run times, multiple short movies (10–20 frames) were collected following the initial “long timelapse” movie (120–180 frames) that was used to measure filament elongation rates. Filaments containing adjacent segments exhibiting dim and bright fluorescence were selected for analysis. We excluded filaments whose ends were not clearly visible in the field of view. We also excluded filaments that visibly stalled and became stuck to the surface. The barbed end of each filament was identified by observing its elongation. A transition from a dim to a bright fluorescence intensity in the barbed end direction indicated the location of formin dissociation. We measured the length of the dim stretch of the filament to determine the formin’s run length (Fig. 3*B*). The number of subunits polymerized by the formin prior to dissociation was calculated by multiplying the run length (in μm) by 370 subunits/μm (9). The formin run length was used to calculate the run time using the following equation:

(3) $$Run\;time\;\left(s\right)=\frac{Number\;of\;steps\;\left(subunits\right)}{Elongation\;rate\;\left(subunits/s\right)}$$

where the elongation rate is determined for each experimental condition as described above. Survival plots were generated by compiling all formin run lengths or run times measured in a particular experimental condition (Figs. 3*C* and 4*B*). A single-exponential fit was applied to each survival curve to obtain the mean run length or the formin off-rate.

### Calculation of probabilities of formin dissociation

To calculate probabilities of dissociation, exponential fits were applied to formin survival curves plotted against the time spent by Bni1p at the barbed end. These fits generated a dissociation rate (*k*_*off-time*_) for each experimental condition. These rates were divided by the experimental elongation rate measured in each condition to obtain a dissociation rate per FH2 step (*k*_*off-step*_). (Although *k*_*off-step*_ values can also be determined by applying fits to survival curves plotted as a function of the lengths of filaments assembled by Bni1p, use of the temporal survival distributions produces higher quality fits, thus enabling more accurate probability measurements.) The probability of formin dissociation per FH2 step (*p*_*off-step*_) was then obtained according to the following equation,

(4) $$p_{off-step}=1-e^{\left(-k_{off-step}*\Delta step\right)}$$

where *k*_*off-step*_ is the formin dissociation rate for a given experimental condition, and *Δstep* is the increment used for the simulation (*i.e.*, 1 FH2 step) (Fig. 5*A*). The probability of formin dissociation per unit time (*p*_*off-time*_) was obtained using our experimentally determined off-rates according to the following equation,

(5) $$p_{off-time}=1-e^{\left(-k_{off-time}*\Delta t\right)}$$

where *k*_*off-time*_ is the formin dissociation rate for a given experimental condition, and *Δt* is the time increment used for the simulation (*i.e.*, 0.01 s) (Fig. 5*B*).

### Simulations of formin-mediated filament assembly

Formin-mediated filament assembly was simulated using experimentally determined parameters and computational programs written using MATLAB (Mathworks) (Fig. 5, *C*–*E*). Each simulation was run for 1200 s using a sample size of 12,000 filaments, each of which was elongated independently. At each simulated time step, a random number between 0 and 1 was generated and compared with either *p*_*off-step*_ or *p*_*off-time*_. If the number exceeded *p*_*off-step*_, the filament was elongated by a single actin subunit. Similarly, if the number exceeded *p*_*off-time*_, the filament was elongated incrementally by a length determined by multiplying the experimentally calculated elongation rate by *Δt* (*i.e.*, 0.01 s). If the number was smaller than *p*_*off-step*_ or *p*_*off-time*_, the formin dissociated and the length of the filament was recorded.

To approximate our experimental conditions, we included a filament nucleation parameter reflecting the number of formin-assembled filaments that are visible in TIRF micrographs collected over the course of our experimental reactions (Fig. S3). Each simulation began with an initial pool of 2400 filaments (*i.e.*, 20% of the total number of filaments). Additional filaments were allowed to nucleate at a constant rate of 8 filaments/s over the course of each simulated polymerization reaction. This nucleation rate was determined by quantifying the number of filaments that were visible in TIRF micrographs collected over the course of our experimental reactions and normalizing this value to the total number of filaments in our simulations (*i.e.*, 12,000) (Fig. S3) (58).

Simulations of actin assembly mediated by a formin employing a range of combinations of FH1-mediated delivery and direct subunit binding (Fig. 9*B*) were performed using experimental measurements of the fraction of polymerized actin subunits that were incorporated by *via* Bni1p′s FH1 domain (See “Calculations of FH1-mediated delivery during elongation”, below) (Fig. 7*F*). A linear fit was applied to extrapolate dissociation rates in conditions in which more than two-thirds of the polymerized actin subunits are incorporated *via* FH1-mediated delivery. Probabilities of formin dissociation per unit time were calculated using these dissociation rates and Equation 5, as described above. The average basal dissociation rate displayed by Bni1p FH1FH2-C (*i.e.*, 0.0039 s^−1^) was employed to calculate the probability of formin dissociation in simulations sampling polymerization below the two-thirds threshold in FH1 efficiency. These probability measurements were applied to simulate actin polymerization at a range of elongation rates using a sample size of 12,000 filaments and a reaction time of 1200 s.

### Calculations of FH1-mediated delivery during elongation

For every polymerization reaction, the fluorescence intensities of formin-assembled segments of filaments were normalized to the intensity of a segment of the same filament generated following the dissociation of the formin. The fraction of unlabeled actin subunits polymerized *via* FH1-mediated delivery (*FH1*_*Black*_) was then calculated using the following equation,

(6) $${FH1}_{Black}=1-{Fluorescence}_{Formin-bound}$$

where *Fluorescence*_*Formin-bound*_ is the normalized fluorescence intensity of the formin-assembled filament segments in the reaction. The fraction of Oregon Green-labeled actin monomers polymerized *via* FH1-mediated delivery (*FH1*_*Green*_) was calculated using the following equation,

(7) $${FH1}_{Green}={FH1}_{Black}\times \frac{GreenPA}{BlackPA}$$

where *Green* *profilin–actin* and *Black* *profilin–actin* are the fraction of profilin-bound actin that are labeled and unlabeled, respectively. These values were calculated using published affinities of *Sc* and *Hs* profilin for unlabeled and labeled actin monomers (2.9 and 29 μM for *Sc* profilin, and 0.1 and 1 μM for *Hs* profilin (30, 39)). The total fraction of polymerized actin subunits that were delivered by the FH1 domain was calculated by summing *FH1*_*Black*_ and *FH1*_*Green*_ (Fig. 7*F*) (9).

### Statistical analysis and error calculations

Statistical errors in mean formin run lengths and dissociation rates were obtained from 95% confidence intervals associated with exponential fits applied to the data. Statistical comparisons of mean run length values were performed using Kruskal–Wallis tests with the level of significance set at 0.05. Statistical comparisons of dissociation rates were performed using extra sum of squares F-tests to compare the exponential fits applied to the survival plots. The level of significance was set at 0.05 to differentiate significantly dissimilar formin dissociation rates.

## Data availability

All data required for the conclusions made here are contained within the article or Supporting information. Any other data will be shared by the corresponding author upon request. The software codes for the simulation programs are available at the following DOI: https://doi.org/10.5281/zenodo.14201504.

## Supporting information

This article contains supporting information.

## Conflict of interest

The authors declare that they have no conflicts of interest with the contents of this article.

## References

[1] Valencia F.R., Sandoval E., Du J., Iu E., Liu J., Plotnikov S.V. (2021). Force-dependent activation of actin elongation factor mDia1 protects the cytoskeleton from mechanical damage and promotes stress fiber repair. Dev. Cell..

[2] Goode B.L., Eck M.J. (2007). Mechanism and function of formins in the control of actin assembly. Annu. Rev. Biochem..

[3] Oosterheert W., Boiero Sanders M., Funk J., Prumbaum D., Raunser S., Bieling P. (2024). Molecular mechanism of actin filament elongation by formins. Science.

[4] Palmer N.J., Barrie K.R., Dominguez R. (2024). Mechanisms of actin filament severing and elongation by formins. Nature.

[5] Vavylonis D., Kovar D.R., O’Shaughnessy B., Pollard T.D. (2006). Model of formin-associated actin filament elongation. Mol. Cell..

[6] Courtemanche N. (2018). Mechanisms of formin-mediated actin assembly and dynamics. Biophys. Rev..

[7] Pimm M.L., Hotaling J., Henty-Ridilla J.L. (2020). Profilin choreographs actin and microtubules in cells and cancer. Int. Rev. Cell Mol. Biol..

[8] Kovar D.R., Harris E.S., Mahaffy R., Higgs H.N., Pollard T.D. (2006). Control of the assembly of ATP- and ADP-actin by formins and profilin. Cell.

[9] Sherer L.A., Zweifel M.E., Courtemanche N. (2018). Dissection of two parallel pathways for formin-mediated actin filament elongation. J. Biol. Chem..

[10] Paul A.S., Pollard T.D. (2008). The role of the FH1 domain and profilin in formin-mediated actin-filament elongation and nucleation. Curr. Biol..

[11] Courtemanche N., Pollard T.D. (2012). Determinants of formin Homology 1 (FH1) domain function in actin filament elongation by formins. J. Biol. Chem..

[12] Funk J., Merino F., Venkova L., Heydenreich L., Kierfeld J., Vargas P. (2019). Profilin and formin constitute a pacemaker system for robust actin filament growth. Elife.

[13] Zweifel M.E., Courtemanche N. (2020). Profilin’s affinity for formin regulates the availability of filament ends for actin monomer binding. J. Mol. Biol..

[14] Zigmond S.H., Evangelists M., Boone C., Yang C., Dar A.C., Sicheri F. (2003). Formin leaky cap allows elongation in the presence of tight capping proteins. Curr. Biol..

[15] Kovar D.R., Wu J.-Q., Pollard T.D. (2005). Profilin-mediated competition between capping protein and formin Cdc12p during cytokinesis in fission yeast. Mol. Biol. Cell..

[16] Paul A.S., Pollard T.D. (2009). Energetic requirements for processive elongation of actin filaments by FH1FH2-formins. J. Biol. Chem..

[17] Cao L., Kerleau M., Suzuki E.L., Wioland H., Jouet S., Guichard B. (2018). Modulation of formin processivity by profilin and mechanical tension. Elife.

[18] Bremer K.V., Wu C., Patel A.A., He K.L., Grunfeld A.M., Chanfreau G.F. (2024). Formin tails act as a switch, inhibiting or enhancing processive actin elongation. J. Biol. Chem..

[19] Vizcarra C.L., Bor B., Quinlan M.E. (2014). The role of formin tails in actin nucleation, processive elongation, and filament bundling. J. Biol. Chem..

[20] Chesarone M., Gould C.J., Moseley J.B., Goode B.L. (2009). Displacement of formins from growing barbed ends by Bud14 is critical for actin cable architecture and function. Dev. Cell..

[21] Chesarone M.A., DuPage A.G., Goode B.L. (2010). Unleashing formins to remodel the actin and microtubule cytoskeletons. Nat. Rev. Mol. Cell Biol..

[22] Kuhn J.R., Pollard T.D. (2005). Real-time measurements of actin filament polymerization by total internal reflection fluorescence microscopy. Biophys. J..

[23] Schutt C.E., Myslik J.C., Rozycki M.D., Goonesekere N.C.W., Lindberg U. (1993). The structure of crystalline profilin–β-actin. Nature.

[24] Vinson V.K., De La Cruz E.M., Higgs H.N., Pollard T.D. (1998). Interactions of acanthamoeba profilin with actin and nucleotides bound to actin. Biochemistry.

[25] Zweifel M.E., Courtemanche N. (2020). Competition for delivery of profilin–actin to barbed ends limits the rate of formin-mediated actin filament elongation. J. Biol. Chem..

[26] Sept D., Xu J., Pollard T.D., McCammon J.A. (1999). Annealing accounts for the length of actin filaments formed by spontaneous polymerization. Biophys. J..

[27] Zweifel M.E., Sherer L.A., Mahanta B., Courtemanche N. (2021). Nucleation limits the lengths of actin filaments assembled by formin. Biophys. J..

[28] Paul A.S., Pollard T.D. (2009). Review of the mechanism of processive actin filament elongation by formins. Cell Motil..

[29] Kinosian H.J., Selden L.A., Gershman L.C., Estes J.E. (2000). Interdependence of profilin, cation, and nucleotide binding to vertebrate non-muscle actin. Biochemistry.

[30] Eads J.C., Mahoney N.M., Vorobiev S., Bresnick A.R., Wen K.K., Rubenstein P.A. (1998). Structure determination and characterization of Saccharomyces cerevisiae profilin. Biochemistry.

[31] Neidt E.M., Scott B.J., Kovar D.R. (2009). Formin differentially utilizes profilin isoforms to rapidly assemble actin filaments. J. Biol. Chem..

[32] Ezezika O.C., Younger N.S., Lu J., Kaiser D.A., Corbin Z.A., Nolen B.J. (2009). Incompatibility with formin Cdc12p prevents human profilin from substituting for fission yeast profilin. J. Biol. Chem..

[33] Moseley J.B., Goode B.L. (2005). Differential activities and regulation of Saccharomyces cerevisiae formin proteins Bni1 and Bnr1 by Bud6. J. Biol. Chem..

[34] Kubota H., Miyazaki M., Ogawa T., Shimozawa T., Kinosita K., Ishiwata S. (2017). Biphasic effect of profilin impacts the formin mDia1 force-sensing mechanism in actin polymerization. Biophys. J..

[35] Yu M., Yuan X., Lu C., Le S., Kawamura R., Efremov A.K. (2017). mDia1 senses both force and torque during F-actin filament polymerization. Nat. Comm..

[36] Courtemanche N., Lee J.Y., Pollard T.D., Greene E.C. (2013). Tension modulates actin filament polymerization mediated by formin and profilin. Proc. Natl. Acad. Sci..

[37] Jégou A., Carlier M.-F., Romet-Lemonne G. (2013). Formin mDia1 senses and generates mechanical forces on actin filaments. Nat. Comm..

[38] Yu M., Le S., Efremov A.K., Zeng X., Bershadsky A., Yan J. (2018). Effects of mechanical stimuli on profilin- and formin-mediated actin polymerization. Nano Lett..

[39] Courtemanche N., Pollard T.D. (2013). Interaction of profilin with the barbed end of actin filaments. Biochemistry.

[40] Bombardier J.P., Eskin J.A., Jaiswal R., Corrêa I.R., Xu M.Q., Goode B.L. (2015). Single-molecule visualization of a formin-capping protein ‘decision complex’ at the actin filament barbed end. Nat. Comm..

[41] Shekhar S., Kerleau M., Kühn S., Pernier J., Romet-Lemonne G., Jégou A. (2015). Formin and capping protein together embrace the actin filament in a ménage à trois. Nat. Comm..

[42] Higashida C., Kiuchi T., Akiba Y., Mizuno H., Maruoka M., Narumiya S. (2013). F- and G-actin homeostasis regulates mechanosensitive actin nucleation by formins. Nat. Cell Biol..

[43] Higashida C., Miyoshi T., Fujita A., Oceguera-Yanez F., Monypenny J., Andou Y. (2004). Actin polymerization-driven molecular movement of mDia1 in living cells. Science.

[44] Buttery S.M., Yoshida S., Pellman D. (2007). Yeast formins Bni1 and Bnr1 utilize different modes of cortical interaction during the assembly of actin cables. Mol. Biol. Cell..

[45] Evangelista M., Pruyne D., Amberg D.C., Boone C., Bretscher A. (2002). Formins direct Arp2/3-independent actin filament assembly to polarize cell growth in yeast. Nat. Cell Biol..

[46] Kamasaki T., Arai R., Osumi M., Mabuchi I. (2005). Directionality of F-actin cables changes during the fission yeast cell cycle. Nat. Cell Biol..

[47] McInally S.G., Reading A.J.B., Rosario A., Jelenkovic P.R., Goode B.L., Kondev J. (2024). Length control emerges from cytoskeletal network geometry. PNAS.

[48] Baker J.L., Courtemanche N., Parton D.L., McCullagh M., Pollard T.D., Voth G.A. (2015). Electrostatic interactions between the Bni1p formin FH2 domain and actin influence actin filament nucleation. Structure.

[49] Bradley A.O., Vizcarra C.L., Bailey H.M., Quinlan M.E. (2020). Spire stimulates nucleation by Cappuccino and binds both ends of actin filaments. Mol. Biol. Cell..

[50] Bilancia C.G., Winkelman J.D., Tsygankov D., Nowotarski S.H., Sees J.A., Comber K. (2014). Enabled negatively regulates diaphanous-driven actin dynamics in vitro and in vivo. Dev. Cell..

[51] Wen Y., Eng C.H., Schmoranzer J., Cabrera-Poch N., Morris E.J.S., Chen M. (2004). EB1 and APC bind to mDia to stabilize microtubules downstream of Rho and promote cell migration. Nat. Cell Biol..

[52] Breitsprecher D., Jaiswal R., Bombardier J.P., Gould C.J., Gelles J., Goode B.L. (2012). Rocket launcher mechanism of collaborative actin assembly defined by single-molecule imaging. Science.

[53] Henty-Ridilla J.L., Rankova A., Eskin J.A., Kenny K., Goode B.L. (2016). Accelerated actin filament polymerization from microtubule plus ends. Science.

[54] Walker J.M., Gasteiger E., Hoogland C., Gattiker A., Duvaud S., Wilkins M.R. (2005). The Proteomics Protocols Handbook.

[55] Spudich J.A., Watt S. (1971). The Regulation of Rabbit Skeletal Muscle Contraction: I. Biochemical studies of the interaction of the tropomyosin-troponin complex with actin and the proteolytic fragments of myosin. J. Biol. Chem..

[56] Schneider C.A., Rasband W.S., Eliceiri K.W. (2012). NIH Image to ImageJ: 25 years of image analysis. Nat. Methods.

[57] Pollard T.D. (2010). A guide to simple and informative binding assays. Mol. Biol. Cell..

[58] Sherer L.A., Mahanta B., Courtemanche N. (2024). Computational tools for quantifying actin filament numbers, lengths, and bundling. Biol. Open.

