# Supplementary material for: The mode of subunit addition regulates the processive elongation of actin filaments by formin
Source: J Biol Chem. 2024 Dec 10;301(1):108071. doi: 10.1016/j.jbc.2024.108071 (PMC11773026; doi:10.1016/j.jbc.2024.108071)
Supplement: Supporting information [file mmc1.docx]

**Supporting Information**

**Figure S1. Filaments initially identified as spontaneously-assembled contain a dim stretch at their pointed end.** The experimental conditions were as follows: 0.75 µM actin monomers (33% Oregon Green-labeled), 2.5 µM *Sc* profilin, and 10 nM Bni1p FH1FH2 in microscopy buffer. The data were collected by TIRF microscopy. (A) Representative micrographs of two filaments containing short stretches of dim fluorescence at their pointed end. The barbed and pointed ends are marked with green and pink arrowheads, respectively. (B) Fluorescence intensity measured along the length of each filament shown in (A) as a function of the distance from the barbed (green data) and pointed (pink data) ends.

**Supporting Figure S2. A variable probability of dissociation per unit time characterizes the processive behavior of Bni1p.** (A) Histogram of formin run lengths generated in simulated polymerization reactions using the value of *p_off-time_* and the formin-mediated elongation rate derived from experimental reactions containing 0.75 µM actin and 5 µM profilin. A single-exponential fit was applied to the data to obtain the mean run length. (B) Dependence of the mean simulated formin run length obtained using calculated values of *p_off-time_* on the rate of formin-mediated filament elongation. Error bars are standard deviations of the mean run lengths, obtained from 10 independent stochastic simulations. Data points are color-coded according to the concentration of profilin that was included in the experimental reactions used to measure the elongation rates. (C) Dependence of the simulated rate of formin dissociation obtained using calculated values of *p_off-time_* on the rate of formin-mediated filament elongation. Standard deviations of the dissociation rates were calculated from 10 independent stochastic simulations. The magnitude of each standard deviation is smaller than the symbols used to represent the dissociation rates. Therefore, error bars are not visible in the graph. Data points are color-coded according to the concentration of profilin that was included in the experimental reactions used to measure the elongation rates.

**Supporting Figure S3. Quantification of formin-mediated nucleation rates.** The experimental conditions were as follows: 0.75 µM actin monomers (33% Oregon Green-labeled) in microscopy buffer with 10 - 25 nM Bni1p FH1FH2 and a range of concentrations of profilin. The number of filaments nucleated by Bni1p was quantified from TIRF micrographs collected over the course of polymerization reactions containing 2.5 µM (circles), 5 µM (squares), and 10 µM (triangles) profilin. Data were normalized to the total number of filaments observed at the final timepoint (i.e., 1200 s) of each reaction. Linear fits were applied to the data and reflect a constant nucleation rate, independent of the profilin concentration. The error bars are standard deviations of the average number of filaments visualized in micrographs collected at each time point in three independent experiments.
